# Supplementary figures and images for: Congenital Chagas disease: A cohort study to assess molecular diagnostic methods at the Chagas disease national reference center of Argentina
Source: PLoS Negl Trop Dis. 2025 Jan 10;19(1):e0012785. doi: 10.1371/journal.pntd.0012785 (PMC11825091; doi:10.1371/journal.pntd.0012785)

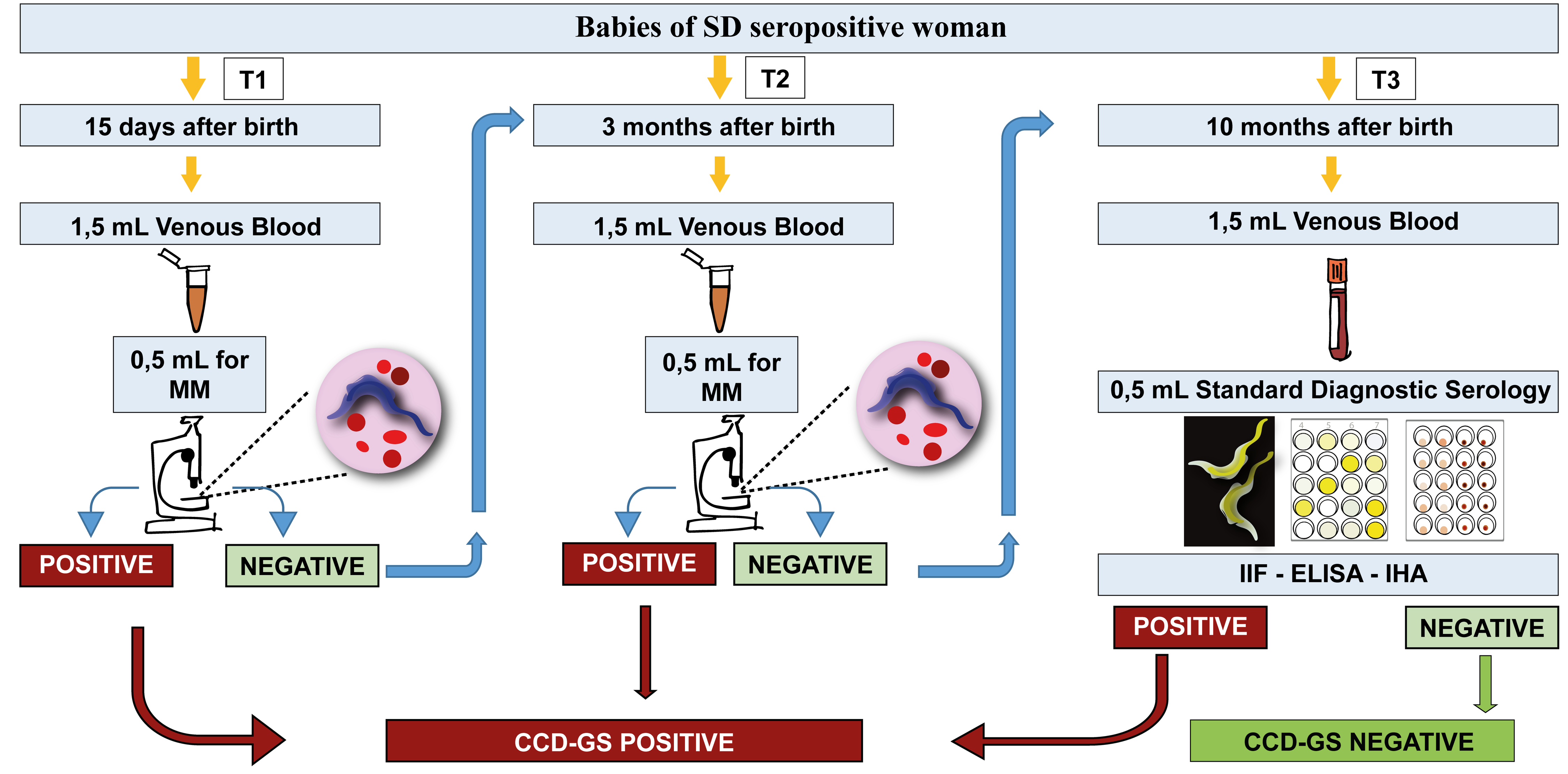

Supplement: S1 Fig — (TIF) [file pntd.0012785.s001.tif]

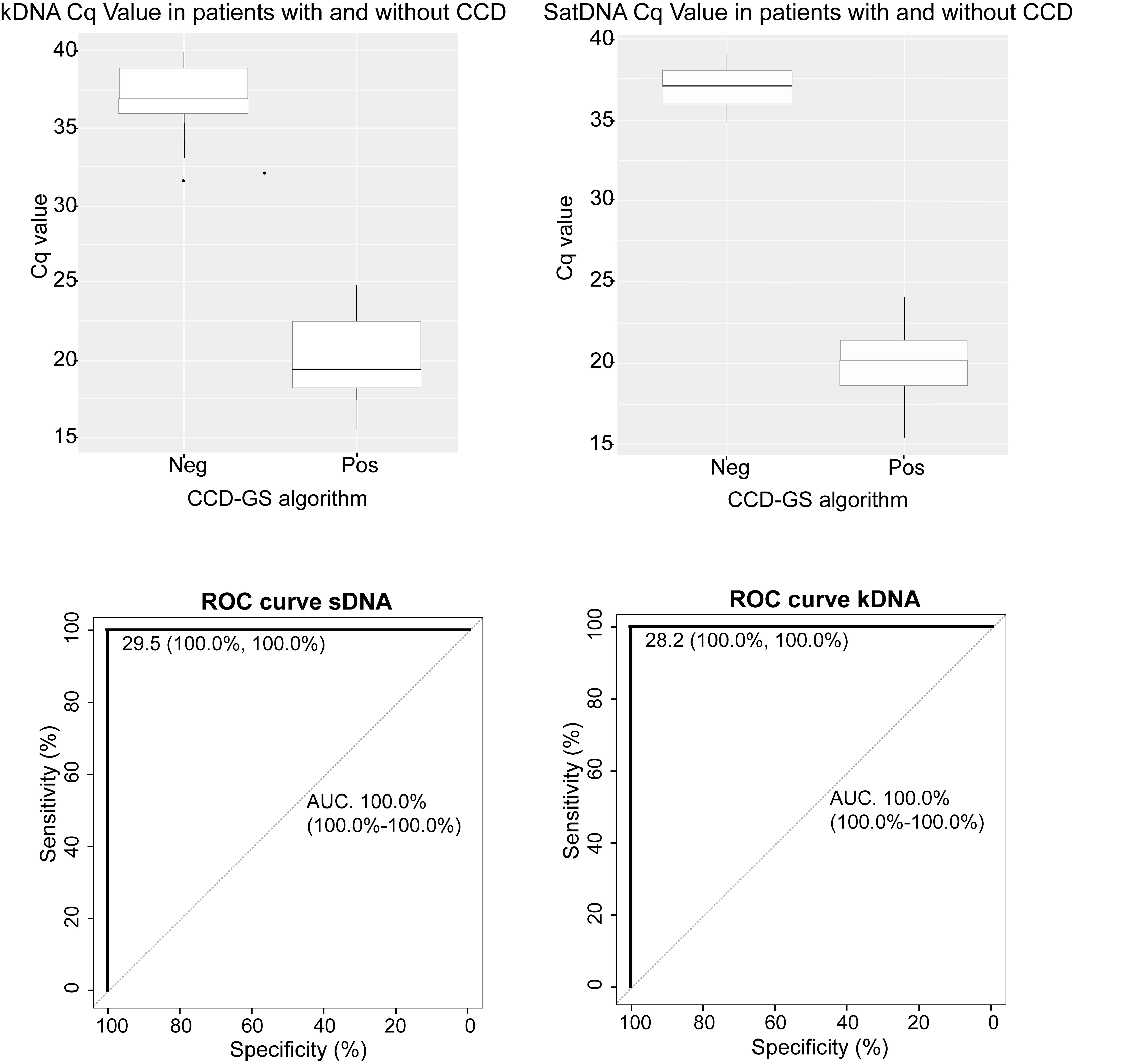

Supplement: S2 Fig — Mario Fatala Chaben (TIF) [file pntd.0012785.s002.tif]
