# Supplementary material for: Congenital Chagas disease: A cohort study to assess molecular diagnostic methods at the Chagas disease national reference center of Argentina
Source: PLoS Negl Trop Dis. 2025 Jan 10;19(1):e0012785. doi: 10.1371/journal.pntd.0012785 (PMC11825091; doi:10.1371/journal.pntd.0012785)
Supplement: S1 Written Consent Form — (PDF) [file pntd.0012785.s003.pdf]

ANEXO I

Instituto Nacional de Parasitología "Dr. Mario Fatala Chaben" /

ANLIS "Dr. Carlos G Malbrán"

FORMA DE CONSENTIMIENTO

Título: "*Estudio de Implementación para la transferencia de metodologías Moleculares para el diagnóstico de Chagas congénito al Sistema Sanitario"*

Investigador Principal: Constanza Lopez-Albizu

Director del Proyecto: Carolina Cura

Responsable del Departamento: Bioq.Karenina Scollo

Estimada Sra.:

La estamos invitando a participar en un trabajo de investigación. En este proyecto estamos estudiando si podemos saber al momento del nacimiento si su bebé sufrió la infección congénita con el parásito de la enfermedad de Chagas (*Trypanosoma cruzi*) utilizando pruebas microscópicas, moleculares y serológicas. Se utilizarán los datos de los análisis realizados a 500 hijos de madres con serología reactiva para Enfermedad de Chagas

Procedimientos:

Por los controles que hizo durante el embarazo ya se había diagnosticado que usted es portadora del parásito de la enfermedad de Chagas (*Trypanosoma cruzi*), entonces se harán en su bebé todos los controles que actualmente están indicados por los médicos del hospital. Para el estudio utilizaremos los datos de los resultados de los análisis realizados a su bebé durante todo el seguimiento. Estos datos serán utilizados de forma anónima y el producto de su análisis podría contribuir a mejorar el diagnóstico de Chagas congénito haciéndolo más corto y eficiente.

Riesgos potenciales:

No existen Riesgos potenciales dado que sólo utilizaremos los datos de los resultados de los análisis que le hemos realizado o realicemos a su hijo y de forma anónima.

Riesgos para productos o recién nacidos:

El estudio propuesto no tiene riesgos o éste es mínimo para su bebé.

Beneficios potenciales:

Este estudio no beneficiará a usted ni a su bebé, realizaremos los análisis de rutina que su hijo requiere para ser diagnosticado.

Participación voluntaria:

La participación en la investigación autorizando a usar los datos es voluntaria. Usted puede elegir participar o no. Si elige participar, pero posteriormente no desea hacerlo, usted puede retirarse del estudio en cualquier momento. Si decide no participar o retirarse del estudio, esto no le producirá ningún problema o pérdida de beneficios a que usted o su hijo tenga derecho. Participar en el estudio es gratuito.

Confidencialidad:

Se le proporcionará cualquier nueva información que se obtenga durante el curso del estudio, que pueda afectar su buena disposición para participar. Reforzaremos las medidas para que los resultados de los análisis sean privados y confidenciales.

Cuando se reporten los resultados, todos los datos de identificación serán solo agregados en el hospital. Si usted tiene un análisis positivo para anticuerpos contra el parásito que causa la enfermedad de Chagas se agregará a su historia clínica y a la de su bebé, en el hospital.

Si los resultados del estudio fueran publicados, usted no será identificada por su nombre u otra información que la identifique.

Costos/Pagos:

El participar en este estudio no genera ningún tipo de costo económico para usted ni usted percibirá retribución económica

Preguntas

Si tiene preguntas acerca de esta investigación puede llamar a las Bioquímicas Constanza Lopez Albízu o Carolina Cura al 011- 43317732 del INP Fatala Chaben, o a la Dra. Adelina Riarte al 011-4331-4010 del Comité de Ética del INP Fatala Chaben, ambos en Buenos Aires.

He leído esta forma de consentimiento informado y voluntariamente acepto participar en esta investigación.

____ ____________

Firma y aclaración del autorizante Fecha

____ ____________

Padre/ Representante legalmente autorizado (si corresponde) Fecha

____ ____________

Persona que obtiene el consentimiento Fecha

No puedo leer esta forma de consentimiento, pero me la leyó y explicó ____ (nombre del lector). Acepto voluntariamente participar en esta investigación. También autorizo a usar Información de Salud Protegida como se describe en esta forma de consentimiento.

____ ____________

Sujeto DNI Fecha

____ ____________

Testigo Fecha

____ ____________

Persona que obtiene el consentimiento Fecha

Instituto Nacional de Parasitología "Dr. Mario Fatala Chaben" /

ANLIS "Dr. Carlos G Malbrán"

Informed Consent Form

Title: Implementation Study for the Transfer of Molecular Methodologies for the Diagnosis of Congenital Chagas Disease to the Healthcare System

Principal Investigator: Constanza Lopez-Albizu

Project Director: Carolina Inés Cura

Department Head: Bioq. Karenina Scollo

Dear Madam:

We are inviting you to participate in a research study. In this project, we are studying whether we can determine at the time of birth if your baby has suffered from congenital infection with the Chagas disease parasite (*Trypanosoma cruzi*) using microscopic, molecular, and serological tests. The data from the analyses performed on 500 children of mothers with reactive serology for Chagas Disease will be used.

Procedures:

Since it has already been diagnosed during your pregnancy that you are a carrier of the Chagas disease parasite (*Trypanosoma cruzi*), all the tests that are currently indicated by the hospital doctors will be performed on your baby. For the study, we will use the data from the results of the tests performed on your baby throughout the follow-up. This data will be used anonymously and the product of its analysis could contribute to improving the diagnosis of congenital Chagas, making it shorter and more efficient.

Potential Risks:

There are no potential risks since we will only use the data from the results of the tests that we have performed or will perform on your child and in an anonymous manner.

Risks to Products or Newborns:

The proposed study poses no risks or minimal risks to your baby.

Potential Benefits:

This study will not benefit you or your baby; we will perform the routine tests that your child requires to be diagnosed.

Voluntary Participation:

Participation in the research authorizing the use of data is voluntary. You may choose to participate or not. If you choose to participate but later do not wish to do so, you may withdraw from the study at any time. If you decide not to participate or withdraw from the study, this will not cause any problems or loss of benefits to which you or your child are entitled. Participation in the study is free.

Confidentiality:

You will be provided with any new information obtained during the course of the study that may affect your willingness to participate. We will reinforce measures to ensure that the results of the analyses are private and confidential.

When the results are reported, all identifying data will only be aggregated in the hospital. If you have a positive test for antibodies against the parasite that causes Chagas disease, it will be added to your medical record and that of your baby, at the hospital. If the results of the study were published, you would not be identified by your name or other identifying information.

Costs/Payments:

Participating in this study does not generate any economic cost for you, nor will you receive any financial compensation.

Questions:

If you have any questions about this research, you can call Biochemists Constanza Lopez Albízu or Carolina Cura at 011-43317732 of the INP Fatala Chaben, or Dr. Adelina Riarte at 011-4331-4010 of the Ethics Committee of the INP Fatala Chaben, both in Buenos Aires.

I have read this informed consent form and voluntarily agree to participate in this research.

____ _____________

Authorized person's signature and printed name Date

____ _____________

Parent or legal guardian (if applicable) Date

____ _____________

Person obtaining consent Date

"I cannot read this consent form, but it was read and explained to me by ____ (reader's name). I voluntarily agree to participate in this research. I also authorize the use of Protected Health Information as described in this consent form."

____ ______________

Subject ID number Date

____ ______________

Witness's name and signature Date

____ ______________

Person obtaining consent Date
