# Supplementary material for: Congenital Chagas disease: A cohort study to assess molecular diagnostic methods at the Chagas disease national reference center of Argentina
Source: PLoS Negl Trop Dis. 2025 Jan 10;19(1):e0012785. doi: 10.1371/journal.pntd.0012785 (PMC11825091; doi:10.1371/journal.pntd.0012785)
Supplement: S1 Table — Foot table: W: well, S: SatDNAqPCR, k: kDNA qPCR, MM: Parasitological test by Micromethod, SD: standard diagnostic results, R: reactive, NR: no reactive. (DOCX) [file pntd.0012785.s004.docx]

| **T1 sample** | | | | | | | | | **T2 sample** | | | | | | | | | | **T3 sample** | | | | | | | | | |
| --- | --- | --- | --- | --- | --- | --- | --- | --- | --- | --- | --- | --- | --- | --- | --- | --- | --- | --- | --- | --- | --- | --- | --- | --- | --- | --- | --- | --- |
| **Age** | **MM** | **Setep1** | | **Step2** | | | | **Blood parasitic load** | **Age** | **SD** | **MM** | **Step1** | | **Step2** | | | | **Blood parasitic load** | **Age** | **CS** | **MM** | **Setep1** |  | **Step2** |  |  |  | **Blood parasitic load** |
| **(M)** |  | **S** | **k** | **S** | **S** | **k** | **k** |  | **(M)** | **(R/NR)** |  | **S** | **k** | **S** | **S** | **k** | **k** |  | **(M)** | **(R/NR)** |  | **S** | **k** | **S** | **S** | **k** | **k** |  |
|  |  | **w1 (Cq)** | **w1 (Cq)** | **w1 (Cq)** | **w2 (Cq)** | **w1 (Cq)** | **w2 (Cq)** | **par. Eq./mL** |  |  |  | **w1 (Cq)** | **w1 (Cq)** | **w1 (Cq)** | **w2 (Cq)** | **w1 (Cq)** | **w2 (Cq)** | **par. Eq./mL** |  |  |  | **w1 (Cq)** | **w1 (Cq)** | **w1(Cq)** | **w2 (Cq)** | **w1 (Cq)** | **w2 (Cq)** | **par. Eq./mL** |
| 6 | Neg | 15.4 | 18.2 | 16.2 | 16.1 | 15.8 | 15.8 | 17 0576 | 6.9 |  | Neg | 22.2 | 24.7 | 23.4 | 23.5 | 23.7 | 23.7 | 1237 | 10 | R | Neg |  |  |  |  |  |  |  |
| 2.1 | Neg | 22.4 | 24.1 | 22.9 | 23.4 | 21.7 | 21.6 | 2 237 | 4.2 |  | Pos | 19.8 | 21.0 | 21.7 | 21.9 | 19.8 | 19.9 | 4459 |  |  |  |  |  |  |  |  |  |  |
| 1.1 | Neg | 23.9 | 24.7 | 24.8 | 25.7 | 23.8 | 23.7 | 567 | 3.3 |  | Pos | 23.9 | 24.7 | 24.8 | 25.7 | 23.81 | 23.7 | 6831 |  | 0 |  |  |  |  |  |  |  |  |
| 3 | Pos | 17.7 | 19.3 | 18.5 | 18.5 | 18.5 | 18.5 | 29 330 |  |  |  |  |  |  |  |  |  |  |  |  |  |  |  |  |  |  |  |  |
| 1.2 | Neg | 20.7 | 23.0 | 22.3 | 22.3 | 22.2 | 22.9 | 2 506 | 7.2 |  | Pos | 25.8 | 24.0 | 24.8 | 25.23 | 23.8 | 23.9 | 528 |  |  |  |  |  |  |  |  |  |  |
| 1.7 | Pos | 21.4 | 19.7 | 20.7 | 20.7 | 19.9 | 20.0 | 1 121 |  |  |  |  |  |  |  |  |  |  |  |  |  |  |  |  |  |  |  |  |
| 2.2 | Pos | 18.7 | 18.0 | 18.6 | 18.8 | 18.1 | 18.2 | 29 692 |  |  |  |  |  |  |  |  |  |  |  |  |  |  |  |  |  |  |  |  |
| 9 | Neg | 20.9 | 18.6 | 21.4 | 21.3 | 18.8 | 18.9 | 7 149 | 12.4 | R |  |  |  |  |  |  |  |  |  |  |  |  |  |  |  |  |  |  |
| 3.7 | Neg | 18.6 | 16.4 | 17.3 | 17.4 | 16.7 | 16.8 | 72 166 | 10.3 | R | Neg |  |  |  |  |  |  |  |  |  |  |  |  |  |  |  |  |  |
| 0.7 | Neg | 20.5 | 18.7 | 20.7 | 20.8 | 19.2 | 19.2 | 8 162 | 5.4 |  | Neg | 21.6 | 21.4 | 22.8 | 22.8 | 21.2 | 21.2 | 2351 | Pos | 24.3 | 23.0 | 23.3 | 23.3 | 20.8 | 20.8 | 21.0 | 20.5 | 1440 |
| 4 | Pos | 19.7 | 18.5 | 19.1 | 19.1 | 16.8 | 16.8 | 27 170 |  |  |  |  |  |  |  |  |  |  |  |  |  |  |  |  |  |  |  |  |
| 1.5 | Neg | 22.07 | 20.0 | 19.4 | 19.4 | 17.5 | 17.5 | 23 559 |  |  | Pos | 24.8 | 18.2 | 19.1 | 19.1 | 17.1 | 17.3 | 31377 |  |  |  |  |  |  |  |  |  |  |
| 3.2 | Pos | 19.8 | 18.1 | 18.4 | 18.4 | 16.5 | 16.5 | 37 437 | 2.6 |  |  |  |  |  |  |  |  |  |  |  |  |  |  |  |  |  |  |  |
| 1.1 | Neg | 21.3 | 22.3 | 20.6 | 20.8 | 19.4 | 19.4 | 8 348 | 2.1 |  | Pos | 20.7 | 19.8 | 20.3 | 20.4 | 19.6 | 19.5 | 244757 |  |  |  |  |  |  |  |  |  |  |
| 4.9 | Pos | 18.4 | 17.4 | 16.8 | 17.8 | 15.8 | 15.8 | 72 548 |  |  |  |  |  |  |  |  |  |  |  |  |  |  |  |  |  |  |  |  |
| 1.8 | Pos | 16.0 | 15.3 | 18.6 | 18.6 | 17.5 | 17.3 | 37 232 |  |  |  |  |  |  |  |  |  |  |  |  |  |  |  |  |  |  |  |  |
| 1.1 | Pos | 24.1 | 24.1 | 23.1 | 23.3 | 24.2 | 24.1 | 11 276 |  |  |  |  |  |  |  |  |  |  |  |  |  |  |  |  |  |  |  |  |
| 2.1 | Pos | 16.2 | 15.6 | 18.0 | 18.2 | 17.7 | 17.7 | 46 792 |  |  |  |  |  |  |  |  |  |  |  |  |  |  |  |  |  |  |  |  |
| 6.4 | Neg | 23.7 | 22.5 | 25.6 | 25.8 | 23.5 | 23.6 | 390 | 7.2 |  | Neg | 25.6 | 24.4 | 27.4 | 27.9 | 20.9 | 21.0 | 101 | 12 | R |  |  |  |  |  |  |  |  |
| 2.3 | Pos | 20.4 | 24.1 | 23.1 | 23.3 | 24.2 | 24.9 | 6 234 |  |  |  |  |  |  |  |  |  |  |  |  |  |  |  |  |  |  |  |  |
| 0.5 | Neg | 18.8 | 19.4 | 18.4 | 18.3 | 19.7 | 20.1 | 29 187 | 3.2 |  | Pos | 21.5 | 23.1 | 21.6 | 21.5 | 23.1 | 23.0 | 2970 |  |  |  |  |  |  |  |  |  |  |
| 6.3 | Pos | 20.0 | 20.3 | 19.8 | 19.8 | 20.4 | 20.6 | 1 627 |  |  |  |  |  |  |  |  |  |  |  |  |  |  |  |  |  |  |  |  |
