# Supplementary material for: Congenital Chagas disease: A cohort study to assess molecular diagnostic methods at the Chagas disease national reference center of Argentina
Source: PLoS Negl Trop Dis. 2025 Jan 10;19(1):e0012785. doi: 10.1371/journal.pntd.0012785 (PMC11825091; doi:10.1371/journal.pntd.0012785)
Supplement: Attachment — Submitted filename: Cover letter.pdf [file pntd.0012785.s005.pdf]

Buenos aires 05 de Diciembre de 2024

Ref. No.: PNTD-D-24-00941

Dear Editor, Laura-Isobel McCall

I am glad to know that our manuscript is potentially acceptable for publication in Plos NTDs. We appreciate the time that you and the reviewer spent looking over our work, and we believe that the comments and suggestions will improve the quality of the presented data.

After an exhaustive review of the Manuscript ID: PNTD-D-24-00941, entitled “Congenital Chagas Disease: A Cohort Study to Assess Molecular Diagnostic Methods at the Chagas Disease National Reference Center of Argentina, we send the modifications following the suggestions issued by the reviewers hoping to be able to fulfil the expectations. Below you will find our own replies and comments to each point. The changed lines were mentioned according to the "Manuscript V2 CC" file.

We look forward to hearing from you regarding our submission. We would be glad to respond to any further questions and comments that you may have.

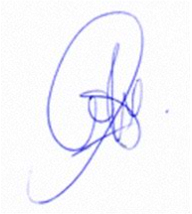

Constanza Lopez-Albizu
